# Supplementary material for: Human Fecal Bile Acid Analysis after Investigational Microbiota-Based Live Biotherapeutic Delivery for Recurrent Clostridioides difficile Infection
Source: Microorganisms. 2023 Jan 5;11(1):135. doi: 10.3390/microorganisms11010135 (PMC9865816; doi:10.3390/microorganisms11010135)
Supplement: Supplementary file 1 [file microorganisms-11-00135-s001.zip › microorganisms-2073644-supplementary.pdf]

Supplementary Information

Human Fecal Bile Acid Analysis after Investigational Microbiota-Based Live Biotherapeutic Delivery for Recurrent *Clostridioides difficile* Infection

Romeo Papazyan [1], Nicky Ferdyan [1], Karthik Srinivasan [1], Carlos Gonzalez [2], Bill Shannon [2], Ken Blount [3] and Bryan C. Fuchs [1,*]

[1] Ferring Research Institute, 4245 Sorrento Valley Blvd, San Diego, CA 92121, USA
[2] BioRankings LLC, St. Louis, MO 63108, USA
[3] Rebiotix Inc., a Ferring Company, Roseville, MN 55113, USA
* Correspondence: bryan.fuchs@ferring.com; Tel.: +1-858-349-4728

The authors declare the following competing interests: CG and BS are consultants of Rebiotix, a Ferring Company.

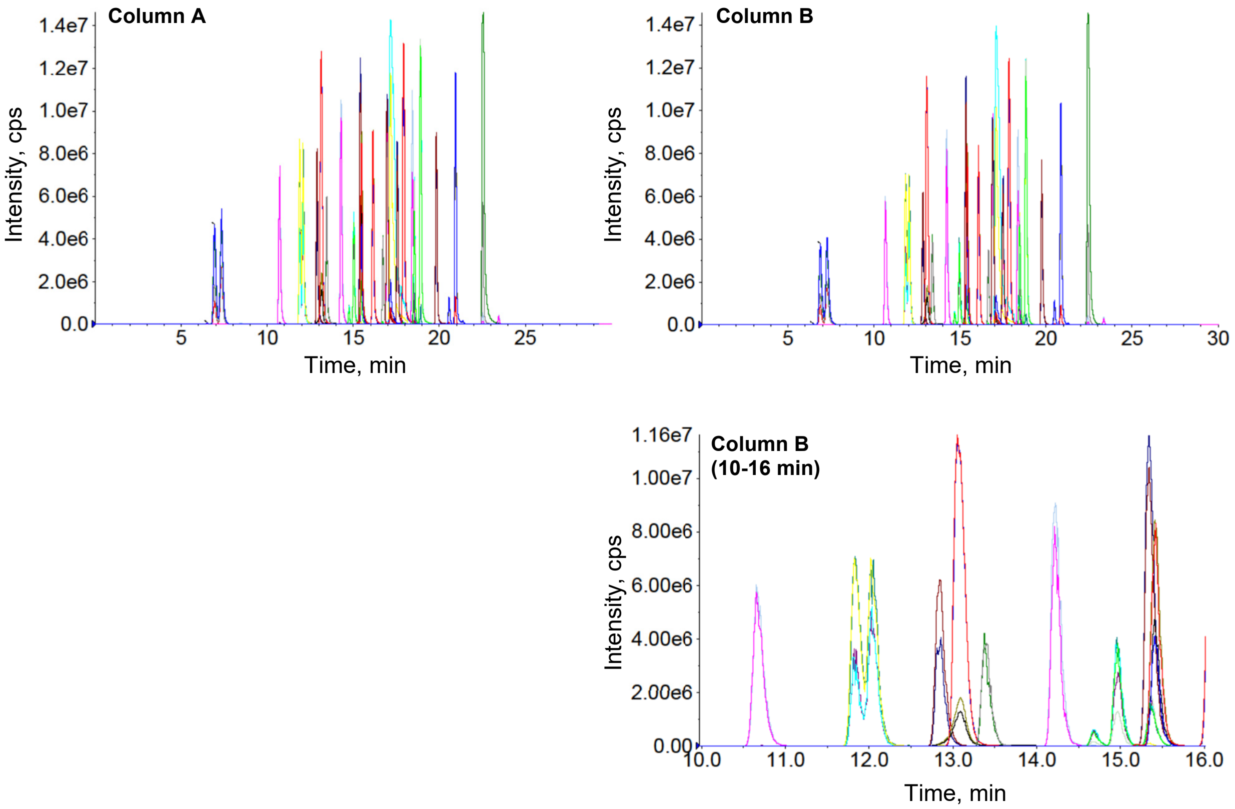

Supplementary Figure S1: Performance of dual column setup**.** Representative MRM chromatograms of BA standards run in parallel using our dual column set up (Column A, Column B). Each BA is represented by a different color. Near identical readouts and column performance was seen throughout study. Zoomed image of a 6 min window on Column B highlights high retention time, reproducibility, and separation fidelity.

(A)

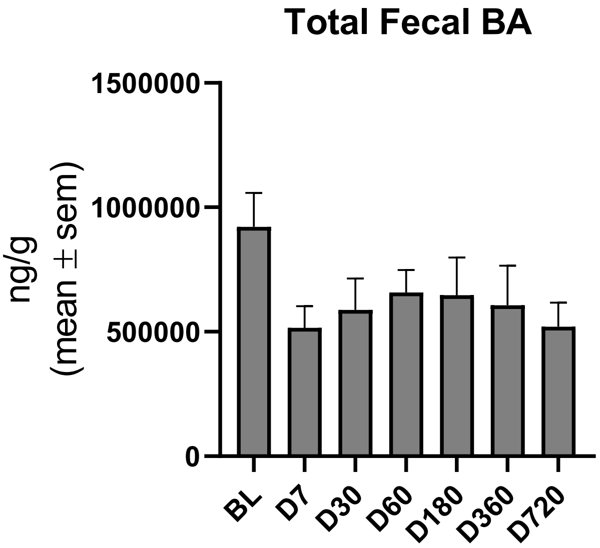

(B)

Relative BA abundance (%) in baseline rCDI patients (n=24)

#	1° Conj	1° Unconj	2° Unconj 7-H	2° Unconj 7-OH /7=O	2° Conj
1	0.1	0.4	95.6	3.7	0.2
2	0.1	0.5	90.8	8.6	0.0
3	2.0	47.5	0.1	50.0	0.4
4	10.4	49.9	0.1	38.7	1.0
5	3.9	64.4	1.8	27.7	2.2
6	29.6	45.0	0.0	15.7	9.6
7	1.0	81.9	1.0	15.9	0.2
8	35.7	47.5	0.0	16.5	0.4
9	0.7	87.5	0.2	10.5	1.1
10	1.2	87.8	0.0	9.9	1.1
11	10.4	78.8	0.0	9.2	1.6
12	2.4	88.3	0.0	8.4	1.0
13	26.6	67.5	0.0	3.5	2.4
14	1.0	94.5	0.0	3.4	1.1
15	15.4	80.4	0.0	1.0	3.2
16	78.7	18.0	0.2	3.1	0.0
17	74.9	22.1	0.0	1.2	1.8
18	27.8	69.6	0.0	0.3	2.3
19	87.0	11.1	0.0	0.7	1.2
20	91.5	6.9	0.0	0.3	1.3
21	15.4	83.4	0.0	0.5	0.8
22	33.1	65.7	0.0	0.3	0.9
23	28.9	70.2	0.0	0.1	0.8
24	98.5	0.9	0.0	0.0	0.6

Supplementary Figure S2: Fecal BA abundance and composition. **(A)**Total fecal BA levels of 35 detected BA (ng BA/g wet fecal matter) from participant samples. **(B)** Percent abundance of primary and secondary BA of baseline patients (each row) broken down by conjugation status and dehydroxylation (7-H), hydroxylation (7-OH), or oxidation (7=O) of the 7-carbon.

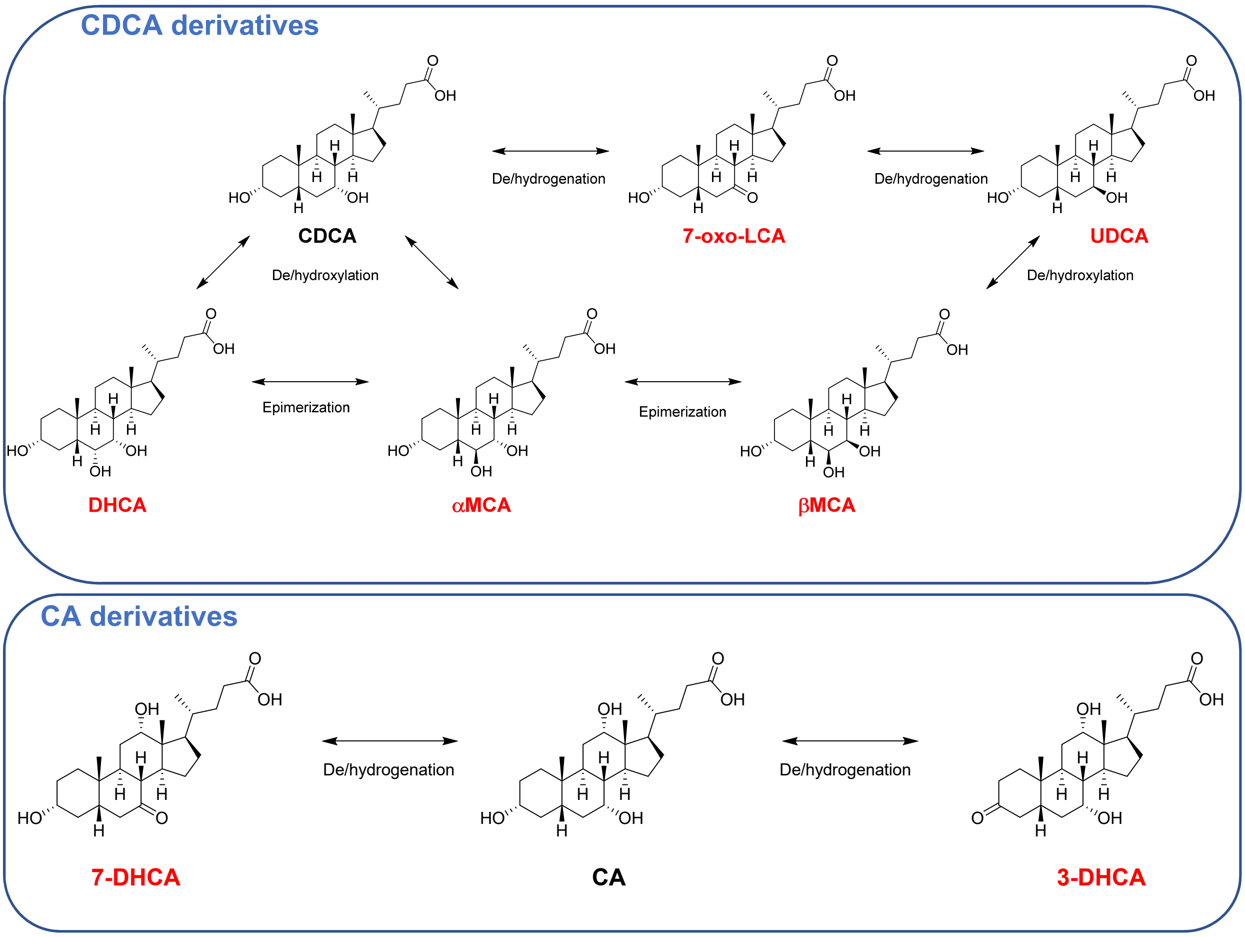

Supplementary Figure S3: Enriched secondary BAs in baseline patients are separated by single biochemical steps**.** Secondary BA enriched in baseline patients are separated by single biochemical steps such as epimerization, dehydroxylation, and dehydrogenation that can be carried out by microbiota. BAs in red are elevated in some baseline rCDI patients.
